# Resolvin D1 attenuates pulmonary hypertension by suppressing pathological connexin 43-mediated gap junctional communication in pulmonary arterial smooth muscle cells

**DOI:** 10.3389/fphar.2026.1940174

**Published:** 2026-10-02

**Authors:** Hong Wang, Nan Wang, Zhixian Ji, Yanhui Zhang, Rui Dong, Silin Pan

**Affiliations:** 1 Heart Center, Qingdao Women and Children’s Hospital, Shandong University, Qingdao, China; 2 Endoscopy Center, Qingdao Municipal Hospital (Qingdao Hospital, University of Health and Rehabilitation Sciences), Qingdao, China; 3 Department of Anesthesiology, Qingdao Municipal Hospital (Qingdao Hospital, University of Health and Rehabilitation Sciences), Qingdao, China

**Keywords:** connexin 43, gap junctions, inflammation, oxidative stress, pulmonary arterial smooth muscle cells, pulmonary hypertension, resolvin D1, vascular remodeling

## Abstract

**Background:**

Pulmonary hypertension (PH) is characterized by progressive pulmonary vascular remodeling driven by activation of pulmonary arterial smooth muscle cells (PASMCs). Resolvin D1 (RvD1), a specialized pro-resolving lipid mediator derived from docosahexaenoic acid (DHA), has vascular protective actions; however, its pharmacological effect on connexin 43 (Cx43)-mediated gap junctional intercellular communication in PH remains unclear. This study investigated whether RvD1 attenuates PH by suppressing pathological formation of gap-junctional Cx43 (GJs-Cx43) in PASMCs.

**Methods:**

SU5416/hypoxia-induced PH was established in C57BL/6 mice. *In vitro*, primary mouse PASMCs were cultured under hypoxic conditions to mimic the hypoxic pathological microenvironment and PASMCs activation associated with PH. Pulmonary hemodynamics, right ventricular function and hypertrophy, pulmonary arterial remodeling, and Cx43 distribution in isolated small pulmonary arteries were evaluated *in vivo*. In PASMCs, biochemical fractionation, Western blotting, immunofluorescence, lucifer yellow dye transfer, cell proliferation and migration assays, and inflammatory and oxidative stress assessments were performed. ZP1609 was used to enhance GJs-Cx43 function *in vitro* and *in vivo*.

**Results:**

RvD1 improved pulmonary hemodynamics and right ventricular adaptation in SU5416/hypoxia-exposed mice. RvD1 also reduced pulmonary arterial wall thickening, elastic lamina remodeling, α-SMA accumulation and PCNA-positive proliferating cells. Mechanistically, SU5416/hypoxia increased total Cx43 and shifted Cx43 toward the GJs-Cx43 formation. RvD1 inhibited GJs-Cx43 formation and attenuated IL-1β, IL-6, ROS and MDA accumulation while partially restoring SOD. In primary PASMCs, hypoxia increased GJs-Cx43 expression, enhanced lucifer yellow dye transfer, and promoted proliferation and migration; these changes were suppressed by RvD1. ZP1609 restored GJs-Cx43-mediated intercellular communication and weakened the inhibitory effects of RvD1 on PASMC inflammatory activation, oxidative stress, proliferation and migration. *In vivo*, ZP1609 similarly blunted the protection conferred by RvD1 against pulmonary vascular remodeling, right ventricular pressure overload and right ventricular hypertrophy.

**Conclusion:**

Pathological GJs-Cx43 formation promotes the spread of remodeling-associated signals across PASMCs networks. RvD1 limits pulmonary vascular remodeling by suppressing Cx43-dependent gap junctional communication, identifying GJs-Cx43 as a pharmacologically relevant mechanism underlying the vascular protection afforded by this DHA-derived lipid mediator.

## 1 Introduction

Pulmonary hypertension (PH) is a debilitating and fatal cardiovascular disorder marked by progressive deterioration of pulmonary arterial function, mainly due to structural remodeling. PH comprises a group of disorders characterized by increased pulmonary vascular resistance, progressive pulmonary vascular remodeling and right ventricular overload (Boucherat O et al., 2025; Olschewski H, 2026). PH is an umbrella term that includes several clinical groups, whereas pulmonary arterial hypertension (PAH) refers specifically to group 1 precapillary PH (Ruopp and Cockrill, 2022). In this study, we use PH to describe the disease phenotype and the Sugen/hypoxia mouse model, and reserve PAH for discussion of the clinical subgroup. Although contemporary therapies improve vasomotor tone and clinical outcomes in selected patients, they do not fully reverse the structural remodeling of small pulmonary arteries (Guignabert C et al., 2024). This persistent remodeling is driven in large part by maladaptive activation of pulmonary arterial smooth muscle cells (PASMCs), which proliferate, migrate and acquire inflammatory and oxidative phenotypes under hypoxic and vascular stress (Boucherat O, et al., 2025). Hypoxia and vascular stress further promote inflammatory and oxidative signaling in PASMCs, thereby sustaining pulmonary vascular remodeling (Bousseau S et al., 2023). Most studies of PASMC remodeling have focused on intracellular signaling pathways within individual cells. However, remodeled pulmonary arteries behave as multicellular networks. Signals generated by a limited number of stressed PASMCs may spread to neighboring cells and synchronize a broader vascular response. This raises an important question: whether pathological intercellular communication itself is a modifiable driver of pulmonary vascular remodeling.

Connexin 43 (Cx43) is the predominant connexin isoform expressed in the lung and vascular cells, where it plays a central role in coordinating intercellular communication through gap junctions (Bouvard C et al., 2020). Functionally, Cx43 exists in two distinct configurations: Cx43 hemichannels and Cx43 gap junctions (GJs-Cx43) (Dong et al., 2022a). Whereas Cx43 hemichannels are less directly involved in cell-to-cell communication, GJs-Cx43 assembles into membrane plaques that mediate gap junctional intercellular communication. Consequently, total Cx43 expression alone does not necessarily reflect the formation or functional activity of communication-competent gap junctions. Under physiological conditions, GJs-Cx43 coordinates tissue homeostasis by enabling direct intercellular signaling. Under pathological conditions, however, excessive GJs-Cx43 may facilitate the propagation of pathological intercellular signals, thereby amplifying inflammatory, oxidative, and proliferative responses across cellular networks (Contreras JE et al., 2004). Emerging evidence has implicated Cx43 in pulmonary vascular reactivity and hypoxia-induced remodeling in PH (Han X-J et al., 2021). However, these studies have largely focused on total Cx43 expression or global Cx43 inhibition, without distinguishing the functional configurations of Cx43. Therefore, whether PH is specifically associated with alterations in GJs-Cx43-mediated intercellular communication remains unknown.

Resolvin D1 (RvD1) is a docosahexaenoic acid-derived specialized pro-resolving mediator that promotes resolution of inflammation and restoration of tissue homeostasis (Alqahtani S et al., 2022; Li X et al., 2024). RvD1 can protect the pulmonary vasculature, including through effects on endothelial-to-mesenchymal transition (Li X, et al., 2024). It remains unclear whether RvD1 protects against PH by regulating the functional configurations of Cx43 toward reduced formation of GJs-Cx43, thereby limiting intercellular communication and preventing the propagation of pathological signals among PASMCs.

Therefore, we hypothesized that RvD1 attenuates pulmonary vascular remodeling by suppressing pathological GJs-Cx43 formation in PASMCs. To test this hypothesis, we evaluated the effects of RvD1 on Cx43 functional configurations, gap junction communication, inflammatory and oxidative signaling, and PASMCs remodeling in experimental PH, and further determined the mechanistic role of GJs-Cx43 using pharmacological restoration of gap junction communication with ZP1609.

## 2 Materials and methods

### 2.1 Animals

Male C57BL/6 mice aged 8 weeks were obtained from Weitong Lihua Experimental Animal Technology Co., Ltd., China. Mice were housed under standard conditions with a 12-h light/dark cycle and free access to food and water. Animal experiments were performed with 8 mice per group (n = 8). All animal procedures were approved by the Laboratory animal Ethics Committee of Nanjing University Medical School Drum Tower Hospital (2021AE01158).

The study was not designed to assess sex-specific responses to RvD1. Male mice were used to maintain a homogeneous cohort in this initial mechanistic study, because sex is a recognized biological modifier of experimental PH (Sun Y et al., 2021). An exploratory pilot study (n = 4 mice per group) was performed before the formal experiment. Using Right ventricular systolic pressure (RVSP) as the primary *in vivo* endpoint and the planned comparison between the H + V and H + RvD1 groups, the estimated sample size was eight mice per group at a two-sided alpha of 0.05% and 80% power. After acclimatization, mice were randomly allocated to experimental groups using a random number table before model induction and treatment.

### 2.2 SU5416/hypoxia-induced PH model and drug administration

Mice were exposed to chronic hypoxia (10% O2) for 3 weeks and received SU5416 (HY-10374, MCE, United States) (20 mg/kg, subcutaneous injection, once weekly) to induce PH. Control mice were maintained in normoxia and received vehicle. RvD1 (10012554, Cayman Chemical, United States) was administered intraperitoneally at 2 μg/kg daily during the 3-week modeling period. For *in vivo* rescue experiments, ZP1609 (GC16618, GlpBio, United States), a rotigaptide-related pharmacological enhancer of Cx43-mediated gap junctional coupling, was administered intraperitoneally at 330 μg/kg every 3 days in RvD1-treated Sugen/hypoxia mice (Dong et al., 2022b).

### 2.3 Echocardiography and hemodynamic measurements

Mice were lightly anesthetized with 1%–1.5% isoflurane (No. H20020,267, Lunan Better Pharmaceutical, China) in oxygen. During the echocardiographic examination, mice were placed in a supine position on a heated platform to maintain body temperature at 37 °C. The isoflurane concentration was carefully titrated to maintain spontaneous breathing and a steady heart rate of 400–500 beats/min to minimize cardiodepressant effects. Pulmonary artery acceleration time/pulmonary ejection time (PAAT/PET) and tricuspid annular plane systolic excursion (TAPSE) were measured to assess pulmonary hemodynamics and right ventricular systolic function. Left ventricular ejection fraction (LVEF) was measured as an index of left ventricular systolic function. Right ventricular systolic pressure (RVSP) was measured immediately after echocardiographic assessment by right heart catheterization. The right external jugular vein was surgically exposed. A heparinized polyethylene catheter (BB31695-PE/1, Smiths medical, UK) was advanced through the right jugular vein into the right ventricle under continuous pressure waveform guidance. RVSP was recorded by Small Animal Monitoring System (ALC-MPA-MS, Shanghai Alcott Biotech, China).

### 2.4 Fulton index and right ventricular morphology

Following hemodynamic measurements, mice were deeply anesthetized with 1% pentobarbital sodium (50 mg/kg, intraperitoneally). Adequate depth of anesthesia was confirmed by absence of the pedal withdrawal reflex. A thoracotomy was then performed, and the pulmonary circulation was flushed with 4 °C PBS through the pulmonary artery. To preserve lung architecture and prevent alveolar collapse, *in situ* lung inflation fixation was performed. The lungs were inflated *via* intratracheal instillation of 4% paraformaldehyde at a constant pressure of 20–25 cm H_2_O. The trachea was then ligated, and the right lung was excised and immersed in 4% paraformaldehyde for histological analysis, while the left lung was stored at −80 °C for biochemical analyses. The right ventricle (RV) was separated from the left ventricle plus septum (LV + S) to determine the Fulton index [RV/(LV + S)]. To avoid tissue disruption from dissection, a separate cohort of mice was utilized for cardiac histology. Transverse cardiac sections were stained with hematoxylin and eosin (HE), and right ventricular cross-sectional wall thickness was quantified by Saiviewer (2.4.0, Servicebio, China).

### 2.5 Lung histology and vascular remodeling analysis

Pulmonary vascular remodeling and pulmonary arterial medial wall thickness (MWT) were assessed on HE and elastin van Gieson (EVG)-stained lung sections. Lung tissues fixed in 4% paraformaldehyde were embedded in paraffin and sectioned at 4 μm thickness. HE staining and elastin van Gieson (EVG) staining were performed according to previously published protocols (Liu G et al., 2021; Weiss A et al., 2019). Small pulmonary arteries (external diameter 25–150 μm) accompanying terminal bronchioles were analyzed. Wall thickness (WT) was defined as the mean distance between the external elastic lamina and the vessel lumen, and medial area is the area between the internal and external elastic laminae. MWT (%) = (2 × WT/external vessel diameter) × 100. For each mouse, five lung sections from different levels were analyzed, and ten vessels were selected from each section. The measurements from 50 vessels within a single mouse were averaged to generate a single biological mean per animal for statistical analysis. All measurements were performed in a blinded manner.

### 2.6 Immunofluorescence staining of lung sections

Paraffin-embedded lung sections were deparaffinized in xylene and rehydrated through graded ethanol. After antigen retrieval, sections were blocked with blocking buffer and incubated overnight at 4 °C with primary antibodies against PCNA(1:500 dilution, 10205-2-AP, Proteintech, China) and α-SMA (1:3,000 dilution, 67735-1-Ig, Proteintech, China). After washing with PBS, sections were incubated with species-appropriate fluorescent secondary antibodies at room temperature in the dark. Nuclei were counterstained with DAPI (P0131, Beyotime, China), and images were captured using Thunder Imaging System (DMi8, Leica, Germany).

For pulmonary vascular morphometry, intrapulmonary muscular arteries were identified by their location within bronchovascular bundles, in close proximity to bronchioles. Small pulmonary arteries with an external diameter of 25–150 μm accompanying terminal bronchioles were analyzed. PASMCs proliferation was evaluated by calculating the percentage of PCNA-positive cells, which was defined as the number of PCNA-positive nuclei divided by the total number of DAPI-stained nuclei within the α-SMA-positive pulmonary arterial walls, multiplied by 100%. The degree of vascular muscularization was represented by the percentage of α-SMA-positive fluorescent area within the vascular cross-sectional area. For each mouse, five lung sections from different levels were analyzed, with ten vessels selected from each section. All quantification was performed in a blinded manner.

### 2.7 Microdissection of mice small pulmonary arteries for protein analysis

Small pulmonary arteries were microdissected from mouse lung tissue for western blot analysis. Briefly, intrapulmonary small arteries were identified according to their anatomic course alongside the airway tree and were carefully separated from surrounding lung parenchyma, bronchi, pulmonary veins and connective tissue using fine forceps and ophthalmic scissors. The second-to fourth-order intrapulmonary muscular arteries, corresponding approximately to vessels with an external diameter of 100–500 μm, were collected for protein analysis.

### 2.8 Preparation of soluble and insoluble fractions of Cx43

Microdissected small pulmonary arteries and primary PASMCs were used for the preparation of total, soluble, and insoluble Cx43 fractions with reference to the literature (Dong et al., 2022b). For total protein extraction, samples were lysed in RIPA buffer containing protease and phosphatase inhibitors on ice. To separate different functional configurations of Cx43, samples were homogenized or lysed in immunoprecipitation buffer containing 1% Triton X-100 and protease/phosphatase inhibitors. After incubation on ice for 30 min, samples were centrifuged at 12,000 rpm for 30 min at 4 °C. The supernatant was collected as the Triton X-100-soluble fraction, representing non-junctional Cx43. The remaining pellet was resuspended in immunoprecipitation buffer containing 1% Triton X-100 and 4 M urea, sonicated, incubated for 30 min, and centrifuged again at 12,000 rpm for 30 min at 4 °C. The resulting supernatant was collected as the Triton X-100-insoluble fraction, representing gap-junction-plaque-enriched Cx43 fraction (GJs-Cx43). Protein concentrations were determined using a BCA assay before western blot analysis. It is important to note that the Triton X-100-insoluble fraction contains gap junction plaques as well as the detergent-resistant polymerized cytoskeletal pool (e.g., polymerized β-tubulin), which is subsequently solubilized by urea treatment and sonication (Beckmann A et al., 2019; Giannakakou P et al., 1997; Musil and Goodenough, 1991).

### 2.9 Western blot analysis

Equal amounts of protein were separated by SDS-PAGE and transferred onto PVDF membranes. After blocking with 5% skim milk and 0.1% Tween 20 in phosphate-buffered saline (PBS), membranes were incubated overnight at 4 °C with primary antibodies against Cx43 (1:16000 dilution, C6219, MilliporeSigma, United States), IL-1β(0.2 μg/mL, ab9722, Abcam, United States), IL-6 (1:1,000 dilution, ab6672, Abcam,United States), and β-tubulin (1:10000 dilution, 66009-1-1g, Proteintech, China). After washing, membranes were incubated with HRP-conjugated secondary antibodies at room temperature. Protein bands were visualized using an enhanced chemiluminescence reagent, and densitometric analysis was performed using ImageJ software (1.53c, NIH, United States) normalized by β-tubulin.

### 2.10 Isolation and culture of primary mouse PASMCs

Primary murine pulmonary artery smooth muscle cells (PASMCs) were isolated and cultured using the literature method (Liu G, et al., 2021). Briefly, six-week-old mice were anesthetized with 1% pentobarbital sodium (50 mg/kg, intraperitoneally) before thoracotomy. Distal pulmonary arteries were dissected. The adventitia, adipose, connective tissues, and endothelial layers were carefully removed. The remaining medial layer of the pulmonary arteries was cut into 1 mm^2^ pieces and placed into cell culture dishes, covered with autoclaved glass coverslips. The explants were cultured in DMEM/F-12 P/S (KGL1201-500, KeyGen, China) supplemented with 20% fetal bovine serum (10100147, Gibco, China) at 37 °C in a humidified 5% CO_2_ incubator. PASMCs at passages 3 to 6 were used for the subsequent experiments. The purity of the primary PASMCs was determined by α-SMA immunofluorescence staining and flow cytometry analysis.

### 2.11 *In vitro* hypoxia and drug treatment

PASMCs were exposed to hypoxia (2% O2) for 48 h RvD1 was administered at 300 nM, and ZP1609 was administered at 1 μg/mL. Cell experiments were performed with six replicates per group.

### 2.12 Immunofluorescence staining of PASMCs

Primary PASMCs grown on coverslips were washed with PBS and fixed with 4% paraformaldehyde for 15 min at room temperature. After permeabilization with 0.1% Triton X-100 in PBS for 15 min, nonspecific binding was blocked with 10% goat serum for 60 min. Cells were then incubated overnight at 4 °C with primary antibodies against Cx43 (1:400 dilution, C6219, MilliporeSigma, United States). After washing with PBS, cells were incubated with appropriate fluorescent secondary antibodies for 90 min at room temperature in the dark, followed by DAPI staining. Fluorescence images were captured using Thunder Imaging System and analyzed with ImageJ software ImageJ software. Cx43-positive puncta with an area ≥1 μm^2^ were quantified as an index of GJs-Cx43 formation. At least three randomly selected fields were analyzed for each group, and all experiments were repeated independently.

### 2.13 Lucifer yellow scrape transfer assay in PASMCs

Gap junctional intercellular communication was assessed using the lucifer yellow scrape transfer assay (Basu R et al., 2015). Primary PASMCs were seeded in culture plates and used when they reached 90%–95% confluence. After the indicated treatments, cells were gently washed with PBS and scraped with a sterile pipette tip to allow Lucifer yellow to enter the cells along the scrape line. Lucifer yellow solution (5 mg/mL, L0259, MilliporeSigma, United States) was then added and incubated at 37 °C for 30 min to allow dye transfer to adjacent cells through gap junctions. Excess dye was removed by washing with PBS, and fluorescence images were captured using a fluorescence microscope. The distance and intensity of Lucifer yellow diffusion from the scrape line were quantified using ImageJ software. Three randomly selected fields were analyzed for each well, and six independent experiments were repeated.

### 2.14 Cell proliferation and migration assays

PASMCs proliferation was assessed using a CCK-8 assay (C0038, Beyotime, China) according to the manufacturer’s instructions. Absorbance was measured at 450 nm. PASMCs migration was assessed using a wound-healing assay. Primary PASMCs were seeded in 6-well plates and cultured until they reached 90%–95% confluence. A linear scratch was generated in the cell monolayer using a sterile 200-μL pipette tip, and detached cells were removed by washing with PBS. Images of the same wounded areas were captured at 0 and 24 h using a light microscope. Wound closure was quantified using ImageJ software and expressed as the percentage of wound area recovered after 24 h.

### 2.15 Oxidative stress assays

Oxidative stress was assessed by measuring Superoxide dismutase (SOD) activity, malondialdehyde (MDA) and reactive oxygen species (ROS) levels. Intracellular ROS levels were detected using the fluorescent probe 2′,7′-dichlorofluorescein diacetate (DCFH-DA) (S0033S, Beyotime, China) according to the manufacturer’s instructions. Briefly, freshly isolated pulmonary artery tissues or primary PASMCs were incubated with DCFH-DA after the indicated treatments, washed to remove excess probe, and analyzed by flow cytometry (AccuriTMC6, BD Biosciences, United States). The fluorescence intensity of oxidized DCF was used to reflect intracellular ROS production.

SOD activity and MDA levels in pulmonary artery tissues and PASMCs were measured using commercial assay kits according to the manufacturers’ protocols (SOD Assay Kit:A001-3, Jiancheng, China; MDA Assay Kit:BC0025, Solarbio, China). SOD activity was used to assess antioxidant capacity, whereas MDA levels were used as an index of lipid peroxidation.

### 2.16 Data normalization

For graphical representation of parameters presented as relative levels or “Normalized to Control” (including Western blot densitometry, morphological wall thickness, relative vascular muscularization, and oxidative stress biochemical assays), data were calculated as relative fold changes. Briefly, raw quantitative values were initially normalized to their respective internal controls (e.g., β-tubulin for Western blotting). The final relative value for each sample was then determined by dividing its individual value by the mean value of the control group (the C + V group), effectively setting the mean of the control group to 1.

### 2.17 Statistical analysis

Data are presented as mean ± SEM. Statistical analyses were performed using SPSS 21.0 software (IBM, United States). Data distribution was assessed using the Kolmogorov–Smirnov test, and homogeneity of variance was evaluated using Levene’s test. For comparisons among multiple groups, one-way ANOVA was performed followed by Bonferroni *post hoc* correction when variances were equal, or Dunnett’s T3 test when variances were unequal. A value of P < 0.05 was considered statistically significant.

## 3 Results

### 3.1 RvD1 improves pulmonary hemodynamics and right ventricular remodeling in PH mice

We first examined whether RvD1 protects against PH in the Sugen/hypoxia mouse model (Figure 1A). Compared with normoxic controls, Sugen/hypoxia markedly reduced PAAT/PET and TAPSE, indicating elevated pulmonary vascular load and impaired right ventricular systolic performance. RvD1 treatment significantly increased PAAT/PET and TAPSE compared with vehicle-treated Sugen/hypoxia mice (Figures 1B–D). Direct hemodynamic assessment further confirmed that RVSP was elevated in PH mice and was reduced by RvD1 treatment (Figure 1E). Consistent with lower right ventricular pressure load, RvD1 reduced Fulton index and attenuated right ventricular wall thickening on HE-stained cardiac sections in PH mice (Figures 1F–H). LVEF did not differ significantly among groups (Supplementary Figure S1), suggesting that the observed phenotype predominantly reflected pulmonary vascular and right ventricular changes rather than global left ventricular systolic dysfunction.

**FIGURE 1:**
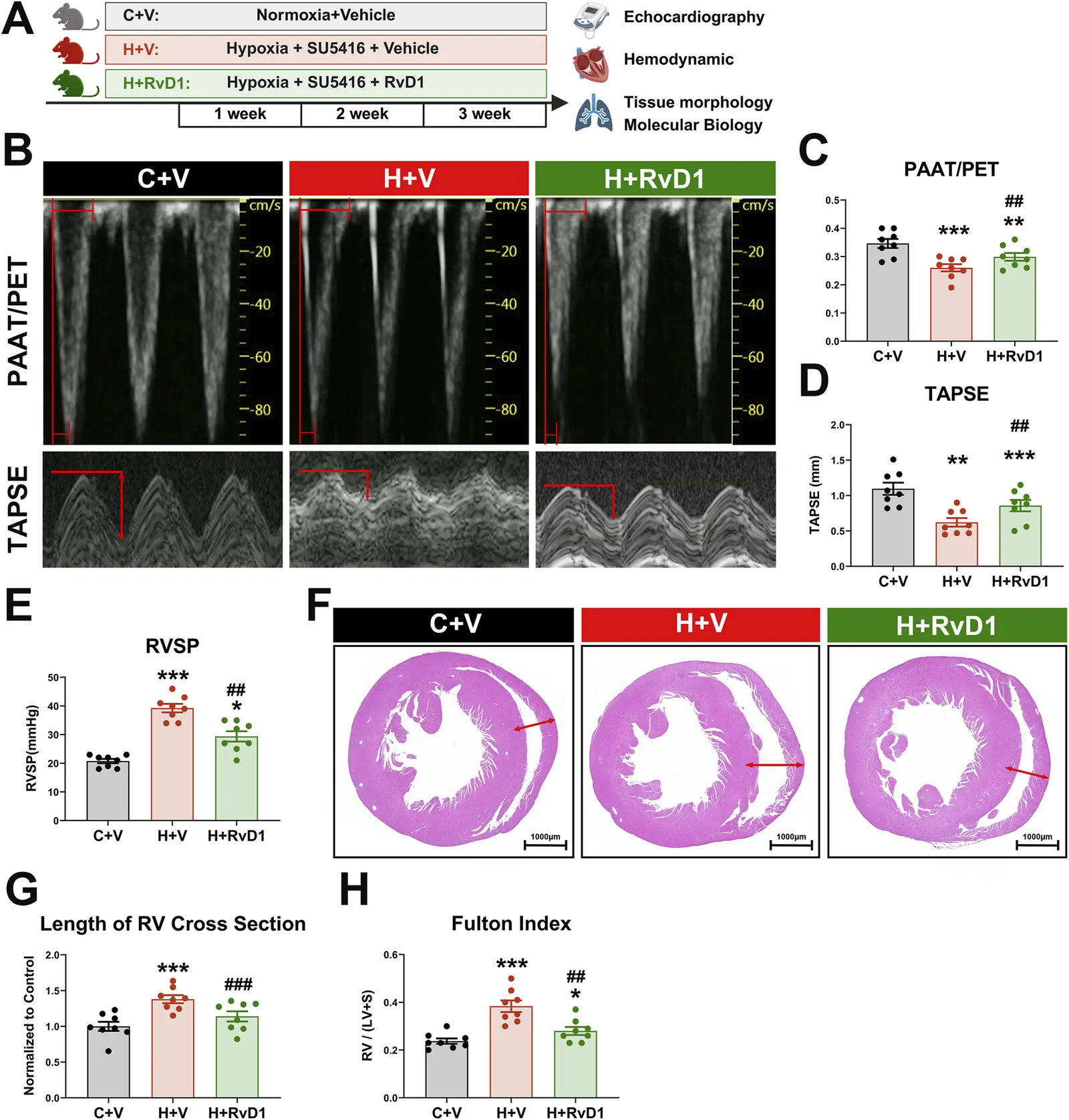
RvD1 improves pulmonary hemodynamics and right ventricular remodeling in PH mice. **(A)** Effects of RvD1 in SU5416/hypoxia-induced PH. Male C57BL/6 mice were randomly allocated to three groups using a random number table: C + V, normoxia plus vehicle; H + V, SU5416/hypoxia plus vehicle; and H + RvD1, SU5416/hypoxia plus RvD1. Mice in the H + V and H + RvD1 groups were exposed to hypoxia (10% O_2_) and treated with SU5416 (20 mg/kg, subcutaneous injection, once weekly) for 3 weeks. C + V mice were maintained under normoxic conditions and received vehicle. RvD1 was administered to the H + RvD1 group at 2 μg/kg by daily intraperitoneal injection throughout the 3-week modeling period. Echocardiography, hemodynamic measurements, tissue morphology, and molecular analyses were performed after completion of the above interventions. **(B)** Representative echocardiographic images showing PAAT/PET and TAPSE measurements in C + V, H + V and H + RvD1 mice. **(C)** Quantification of PAAT/PET. **(D)** Quantification of TAPSE. **(E)** Quantification of Right ventricular systolic pressure (RVSP). **(F)** Representative HE-stained transverse cardiac sections showing right ventricular wall morphology. **(G)** Quantification of right ventricular cross-sectional wall thickness, normalized to the C + V group. **(H)** Quantification of Fulton index. Data are presented as mean ± SEM; n = 8 mice per group. *P < 0.05, **P < 0.01 and ***P < 0.001 *versus* C + V; ^#^ *P* < 0.05, ^##^ *P* < 0.01 and ^###^ *P* < 0.001 *versus* H + V.

### 3.2 RvD1 attenuates pulmonary arterial remodeling and PASMCs proliferation *in vivo*

We next evaluated pulmonary vascular structure by HE and EVG staining. Sugen/hypoxia induced pronounced medial wall thickening and luminal narrowing on HE-stained sections, together with elastic lamina remodeling on EVG-stained sections (Figure 2A). RvD1 substantially reduced MWT quantified from both HE and EVG staining (Figures 2B,C). Immunofluorescence showed increased α-SMA-positive muscularization and PCNA-positive proliferating cells within pulmonary arterial walls after Sugen/hypoxia exposure, whereas RvD1 attenuated both α-SMA expression and PCNA positivity (Figures 2D–F). These findings indicate that RvD1 limits pulmonary arterial muscularization and PASMC proliferative remodeling in PH.

**FIGURE 2:**
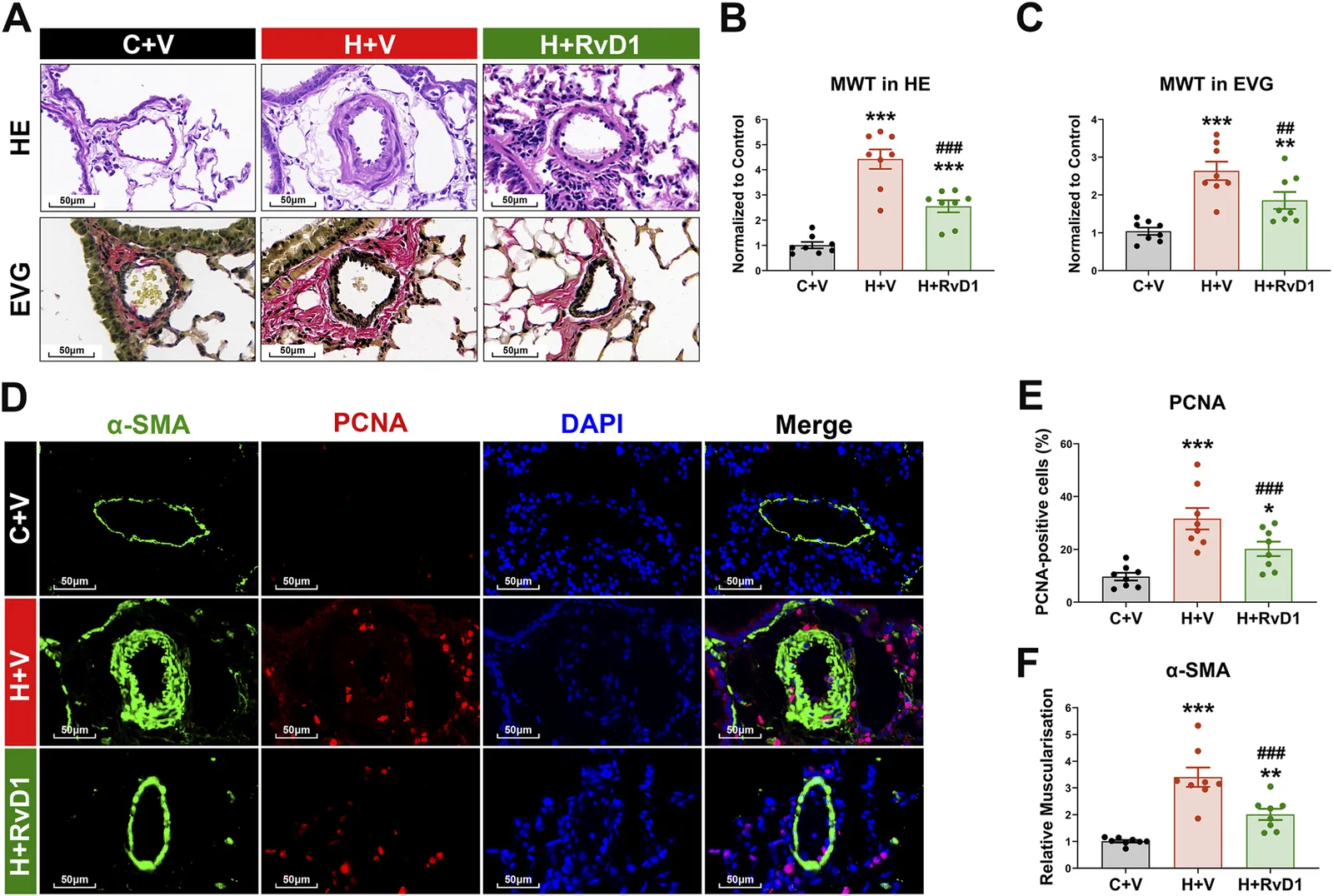
RvD1 attenuates pulmonary arterial remodeling and PASMCs proliferation in PH mice. **(A)** Representative HE and EVG staining of pulmonary arteries from C + V, H + V and H + RvD1 mice. **(B)** Quantification of pulmonary arterial medial wall thickness in HE-stained sections. **(C)** Quantification of pulmonary arterial medial wall thickness in EVG-stained sections. **(D)** Representative immunofluorescence staining for α-SMA, PCNA and DAPI in pulmonary arteries. **(E)** Quantification of PCNA-positive cells in pulmonary arterial lesions. **(F)** Quantification of muscularization by relative α-SMA fluorescence intensity. Data are presented as mean ± SEM; n = 8 mice per group. C + V, normoxia plus vehicle; H + V, Sugen/hypoxia plus vehicle; H + RvD1, Sugen/hypoxia plus RvD1. *P < 0.05, **P < 0.01 and ***P < 0.001 *versus* C + V; ^#^ *P* < 0.05, ^##^ *P* < 0.01 and ^###^ *P* < 0.001 *versus* H + V.

### 3.3 RvD1 suppresses GJs-Cx43 formation and reduces inflammation/oxidative stress in pulmonary arteries from PH mice

Cx43 expression and configuration in microdissected second-to fourth-order intrapulmonary muscular arteries were analyzed. Sugen/hypoxia increased total Cx43 and shifted Cx43 toward the insoluble fraction, representing the GJs-Cx43, while reducing soluble fraction (non-junctional Cx43). RvD1 reduced total Cx43 and GJs-Cx43 while partially restoring soluble/non-junctional Cx43 (Figures 3A,B). These data suggest that PH is associated with preferential GJs-Cx43 formation in pulmonary arteries and that RvD1 inhibits this pathological Cx43 configuration shift.

**FIGURE 3:**
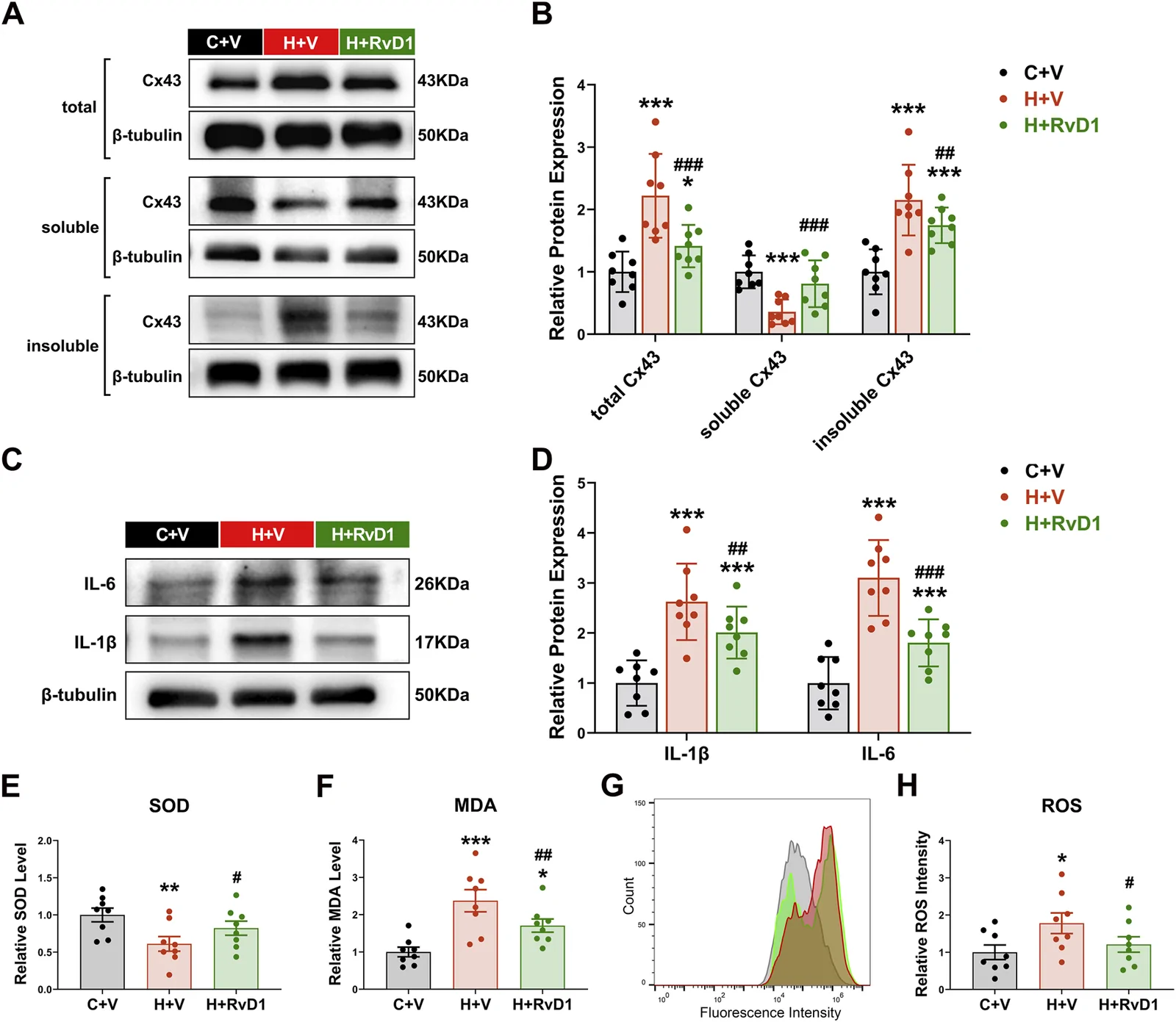
RvD1 suppresses GJs-Cx43 formation, inflammation and oxidative stress in pulmonary arteries from PH mice. **(A)** Representative western blots of total Cx43, soluble Cx43 and insoluble Cx43 in microdissected small pulmonary arteries. **(B)** Quantification of total, soluble and insoluble Cx43 protein expression. **(C)** Representative western blots of IL-1β and IL-6 in small pulmonary artery samples. **(D)** Quantification of IL-1β and IL-6 protein expression. **(E)** Relative SOD level in small pulmonary artery samples. **(F)** Relative MDA level in small pulmonary artery samples. **(G)** Representative flow cytometry histogram showing ROS fluorescence intensity. **(H)** Quantification of relative ROS intensity. Data are presented as mean ± SEM; n = 8 mice per group. C + V, normoxia plus vehicle; H + V, Sugen/hypoxia plus vehicle; H + RvD1, Sugen/hypoxia plus RvD1. *P < 0.05, **P < 0.01 and ***P < 0.001 *versus* C + V; ^#^ *P* < 0.05, ^##^ *P* < 0.01 and ^###^ *P* < 0.001 *versus* H + V.

We then assessed inflammatory and oxidative injury in the same pulmonary arterial samples. Sugen/hypoxia increased IL-1β and IL-6 protein expression, decreased SOD, and elevated MDA and ROS levels. RvD1 reduced IL-1β, IL-6, MDA and ROS while partially restoring SOD (Figures 3C–H). Thus, RvD1-mediated suppression of GJs-Cx43 formation is associated with reduced inflammatory and oxidative stress signaling in remodeled pulmonary arteries from PH mice.

### 3.4 RvD1 suppresses hypoxia-induced GJs-Cx43 formation and primary mice PASMCs proliferation

To determine whether the *in vivo* changes occurred directly in PASMCs, we isolated primary mouse PASMCs and confirmed their purity by α-SMA immunofluorescence and flow cytometry (Supplementary Figure S2). PASMCs were exposed to 2% O_2_ for 48 h and treated with RvD1 at 300 nM (Supplementary Figure S3). Hypoxia increased total Cx43 and insoluble Cx43 while reducing soluble Cx43. RvD1 reduced total and insoluble GJs-Cx43 and partially restored soluble Cx43 (Figures 4A,B), mirroring the *in vivo* findings. Cx43 immunofluorescence showed that hypoxia increased Cx43 puncta larger than 1 μm^2^, consistent with enhanced gap junctions plaque formation, whereas RvD1 reduced Cx43 puncta abunance (Figures 4C,D). Functional GJs-Cx43 was then measured by lucifer yellow scrape dye transfer (Figure 4E). Hypoxia markedly increased dye spread from the scrape line, whereas RvD1 reduced fluorescence propagation and shortened dye transfer distance (Figures 4F–H). Hypoxia also increased PASMCs proliferation as measured by CCK-8 and promoted wound closure in the scratch assay; RvD1 significantly reduced both proliferative activity and migratory wound closure (Figures 4I–K). In addition, RvD1 decreased hypoxia-induced IL-1β and IL-6 expression, partially restored SOD levels, and reduced MDA and ROS accumulation (Figures 4L–Q). Together, these findings show that RvD1 suppresses hypoxia-induced GJs-Cx43 formation and functional gap junctions while limiting PASMC inflammatory activation, oxidative stress, proliferation and migration.

**FIGURE 4:**
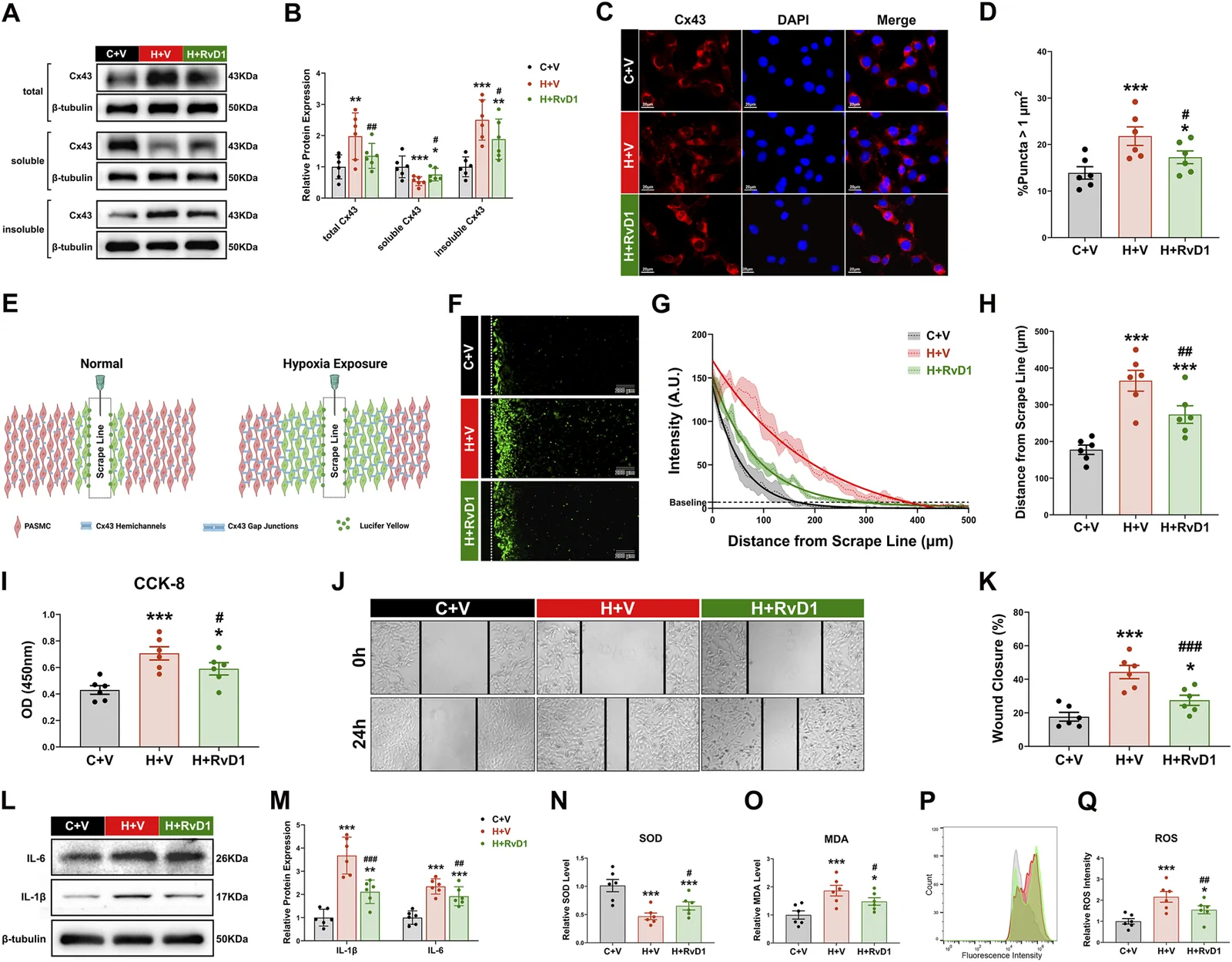
RvD1 suppresses hypoxia-induced Cx43-mediated gap junctional communication and PASMCs activation. **(A)** Representative western blots of total Cx43, soluble Cx43 and insoluble Cx43 in primary mouse PASMCs. **(B)** Quantification of total, soluble and insoluble Cx43 protein expression. **(C)** Representative Cx43 immunofluorescence images in primary mouse PASMCs. **(D)** Quantification of Cx43 puncta larger than 1 μm^2^. **(E)** Schematic diagram of the lucifer yellow scrape dye transfer assay. **(F)** Representative lucifer yellow dye transfer images. **(G)** Fluorescence intensity-distance curves measured from the scrape line. **(H)** Quantification of lucifer yellow transfer distance from the scrape line. **(I)** CCK-8 assay showing PASMC proliferative activity. **(J)** Representative wound healing images at 0 h and 24 h after scratch injury. **(K)** Quantification of wound closure. **(L)** Representative western blots of IL-1β and IL-6 in PASMCs. **(M)** Quantification of IL-1β and IL-6 protein expression. **(N)** Relative SOD level in PASMCs. **(O)** Relative MDA level in PASMCs. **(P)** Representative flow cytometry histogram showing intracellular ROS fluorescence intensity. **(Q)** Quantification of relative ROS intensity. Data are presented as mean ± SEM; n = 6 per group. C + V, normoxia plus vehicle; H + V, hypoxia plus vehicle; H + RvD1, hypoxia plus RvD1. *P < 0.05, **P < 0.01 and ***P < 0.001 *versus* C + V; ^#^ *P* < 0.05, ^##^ *P* < 0.01 and ^###^ *P* < 0.001 *versus* H + V.

### 3.5 ZP1609 restores GJs-Cx43 function and weakens the protective effects of RvD1 in hypoxic PASMCs

To test whether GJs-Cx43 is functionally involved in the protective effect of RvD1, hypoxic PASMCs were treated with ZP1609, a pharmacological enhancer of GJs-Cx43 (Supplementary Figure S4). In RvD1-treated hypoxic PASMCs, ZP1609 increased total Cx43 and insoluble GJs-Cx43 and reduced the RvD1-mediated restoration of soluble/non-junctional Cx43 (Figures 5A,B). ZP1609 also increased Cx43 puncta larger than 1 μm^2^ (Figures 5C,D) and enhanced lucifer yellow dye propagation, as shown by increased fluorescence spread and dye transfer distance (Figures 5E–G). These findings confirm that ZP1609 reactivates GJs-Cx43 under RvD1 treatment.

**FIGURE 5:**
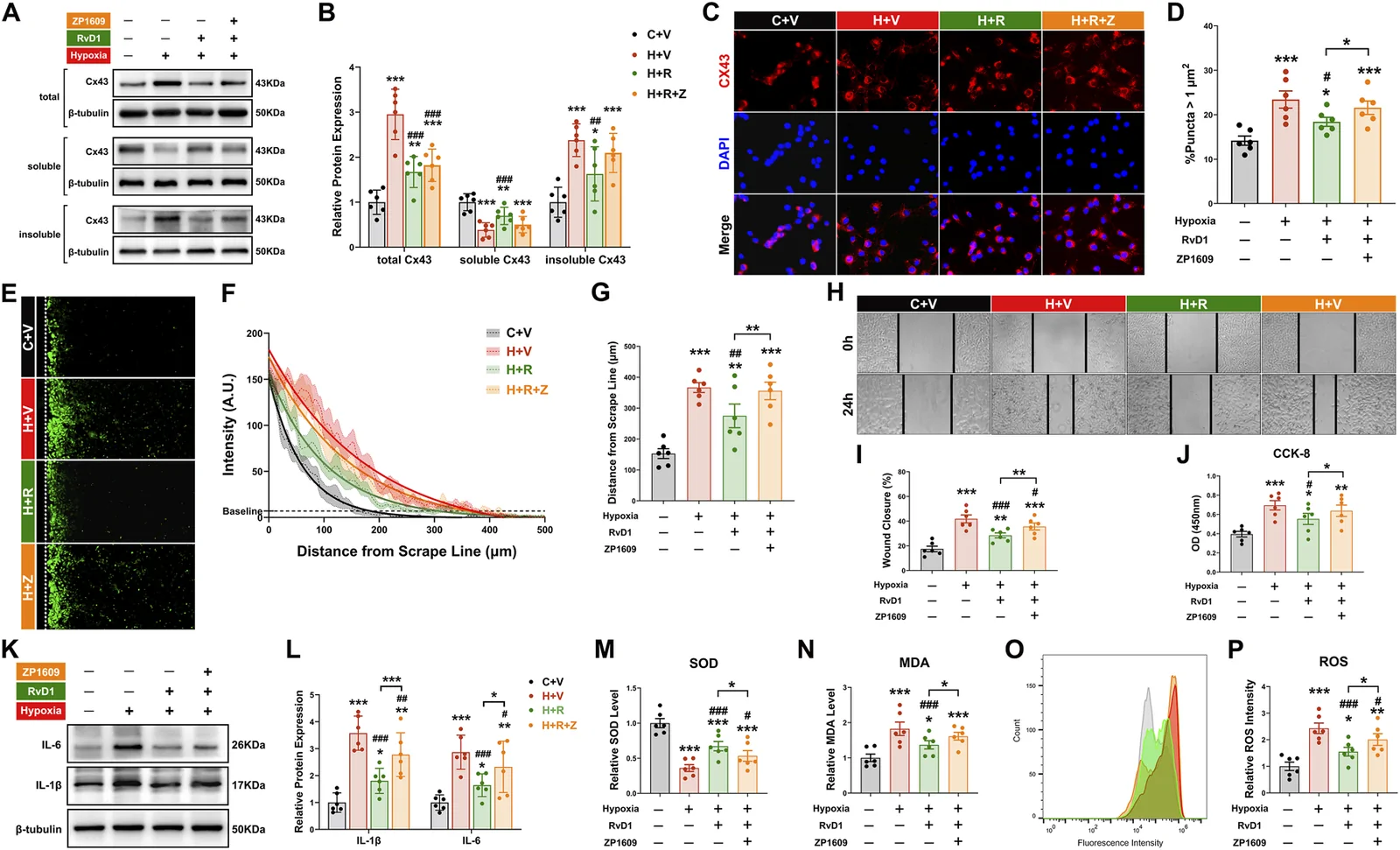
ZP1609 weakens the protective effects of RvD1 in hypoxic PASMCs. **(A)** Representative western blots of total Cx43, soluble Cx43 and insoluble Cx43 in PASMCs treated with hypoxia, RvD1 and ZP1609. **(B)** Quantification of total, soluble and insoluble Cx43 protein expression. **(C)** Representative Cx43 immunofluorescence images. **(D)** Quantification of Cx43 puncta larger than 1 μm^2^. **(E)** Representative lucifer yellow dye transfer images. **(F)** Fluorescence intensity-distance curves measured from the scrape line. **(G)** Quantification of lucifer yellow transfer distance from the scrape line. **(H)** Representative wound healing images at 0 h and 24 h after scratch injury. **(I)** Quantification of wound closure. **(J)** CCK-8 assay showing PASMCs proliferative activity. **(K)** Representative western blots of IL-1β and IL-6 in PASMCs. **(L)** Quantification of IL-1β and IL-6 protein expression. **(M)** Relative SOD level in PASMCs. **(N)** Relative MDA level in PASMCs. **(O)** Representative flow cytometry histogram showing intracellular ROS fluorescence intensity. **(P)** Quantification of relative ROS intensity. Data are presented as mean ± SEM; n = 6 per group. C + V: normoxia plus vehicle; H + V: hypoxia plus vehicle; H + R: hypoxia plus RvD1; H + R + Z: hypoxia plus RvD1 and ZP1609. *P < 0.05, **P < 0.01 and ***P < 0.001 *versus* C + V; ^#^ *P* < 0.05, ^##^ *P* < 0.01 and ^###^ *P* < 0.001 *versus* H + V; brackets indicate comparison between H + R and H + R + Z.

ZP1609 partially reversed the inhibitory effects of RvD1 on PASMC migration and proliferation, increasing wound closure (Figures 5H,I) and CCK-8 absorbance in hypoxic PASMCs (Figure 5J). ZP1609 also blunted the anti-inflammatory and antioxidant effects of RvD1, with increased IL-1β and IL-6 expression, lower SOD levels, and higher MDA and ROS accumulation compared with RvD1-treated hypoxic PASMCs (Figures 5K–P). These data indicate that excessive GJs-Cx43 contributes to the spread of inflammatory, redox and proliferative activation across hypoxic PASMCs networks, and inhibition of GJs-Cx43 is a key component of the anti-inflammatory, antioxidant and anti-proliferative actions of RvD1 in PASMCs.

### 3.6 ZP1609 attenuates the protective effects of RvD1 on pulmonary vascular remodeling, hemodynamics, and right ventricular remodeling *in vivo*

We next evaluated whether enhancement of GJs-Cx43 could weaken the protective effect of RvD1 *in vivo* (Figure 6A). In pulmonary arteries from Sugen/hypoxia mice treated with RvD1, ZP1609 increased total and insoluble GJs-Cx43 and reduced the beneficial shift in soluble Cx43 (Figures 6B,C). ZP1609 also increased IL-1β and IL-6 expression, decreased SOD, and elevated MDA and ROS compared with RvD1 alone (Figures 6D–I). Histological analysis showed that ZP1609 increased pulmonary arterial MWT and elastic lamina remodeling in RvD1-treated PH mice (Figures 6J–L). Immunofluorescence further demonstrated increased α-SMA intensity and PCNA-positive cells after ZP1609 administration (Figures 6M–O). Thus, restoration of GJs-Cx43 counteracts the ability of RvD1 to restrain inflammatory, oxidative and proliferative pulmonary vascular remodeling.

**FIGURE 6:**
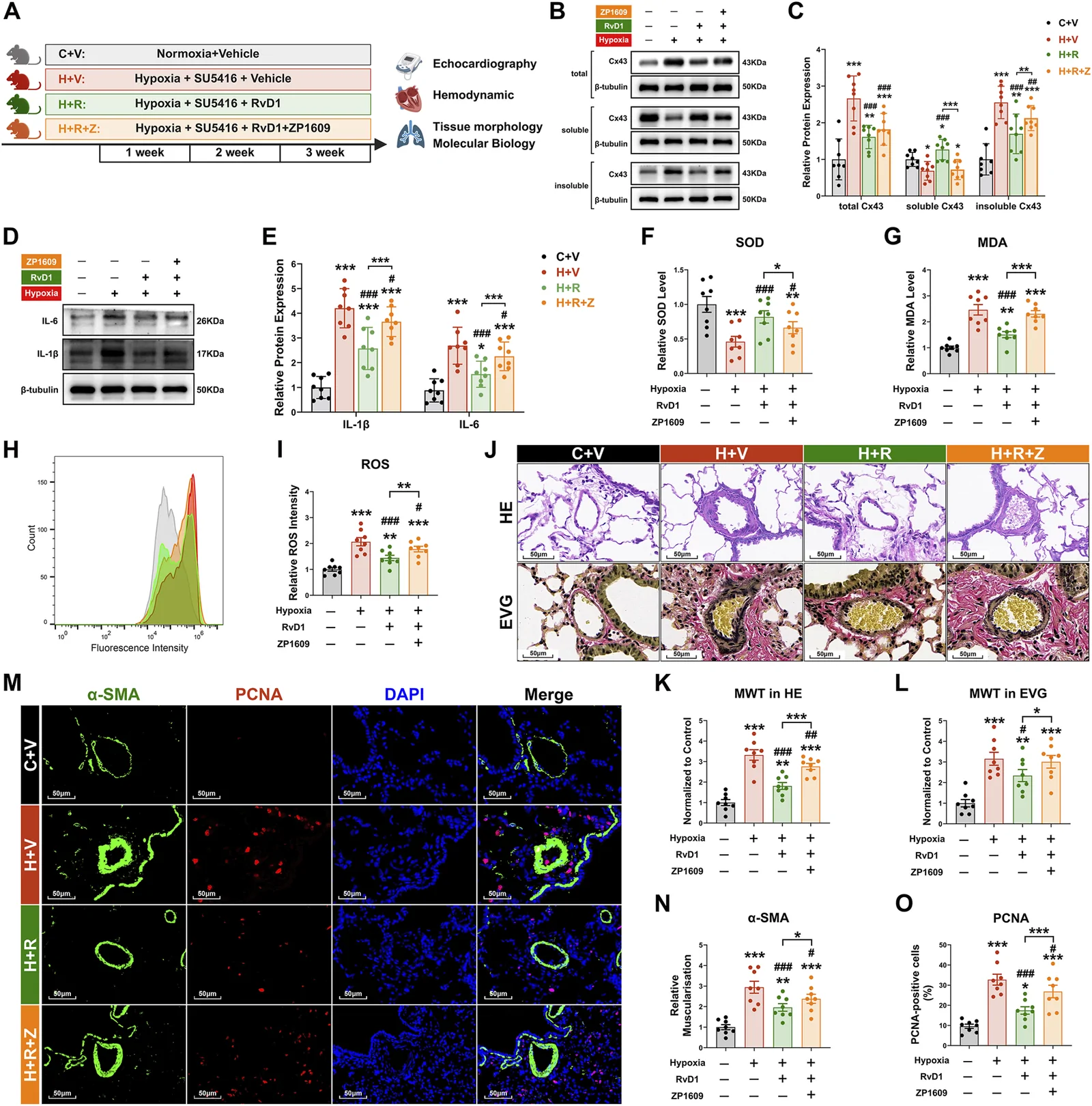
ZP1609 attenuates RvD1-mediated inhibition of pulmonary vascular remodeling in PH mice. **(A)** Experimental design of the *in vivo* ZP1609 intervention study. Male C57BL/6 mice were randomly allocated to four groups using a random number table. C + V mice were maintained under normoxic conditions for 3 weeks and received vehicle. H + V mice were exposed to hypoxia (10% O_2_) for 3 weeks and received SU5416 (20 mg/kg, subcutaneous injection, once weekly) together with vehicle. H + R mice were exposed to hypoxia (10% O_2_) and received SU5416 (20 mg/kg, subcutaneous injection, once weekly) for 3 weeks; in addition, these mice received RvD1 (2 μg/kg, intraperitoneal injection, once daily) throughout the 3-week modeling period. H + R + Z mice received the same hypoxia, SU5416, and RvD1 regimen as the H + R group, with the additional administration of ZP1609 (330 μg/kg, intraperitoneal injection, every 3 days) throughout the modeling period to enhance GJs-Cx43 function. At the end of the 3-week intervention, pulmonary arterial molecular analyses, oxidative-stress assessments, and histological evaluation of pulmonary vascular remodeling were performed. **(B)** Representative western blots of total Cx43, soluble Cx43 and insoluble Cx43 in microdissected small pulmonary arteries. **(C)** Quantification of total, soluble and insoluble Cx43 protein expression. **(D)** Representative western blots of IL-1β and IL-6 in pulmonary artery samples. **(E)** Quantification of IL-1β and IL-6 protein expression. **(F)** Relative SOD level in pulmonary artery samples. **(G)** Relative MDA level in pulmonary artery samples. **(H)** Representative flow cytometry histogram showing ROS fluorescence intensity. **(I)** Quantification of relative ROS intensity. **(J)** Representative HE and EVG staining of pulmonary arteries. **(K)** Quantification of pulmonary arterial medial wall thickness in HE-stained sections. **(L)** Quantification of pulmonary arterial medial wall thickness in EVG-stained sections. **(M)** Representative immunofluorescence staining for α-SMA, PCNA and DAPI in pulmonary arteries. **(N)** Quantification of muscularization by relative α-SMA fluorescence intensity. **(O)** Quantification of PCNA-positive cells in pulmonary arterial lesions. Data are presented as mean ± SEM; n = 8 mice per group. *P < 0.05, **P < 0.01 and ***P < 0.001 *versus* C + V; ^#^ *P* < 0.05, ^##^ *P* < 0.01 and ^###^ *P* < 0.001 *versus* H + V; brackets indicate comparison between H + R and H + R + Z.

Consistent with the vascular remodeling data, ZP1609 weakened the hemodynamic and right ventricular benefits of RvD1 in PH mice. Compared with PH mice treated with RvD1 alone, mice receiving combined RvD1 and ZP1609 showed lower PAAT/PET and TAPSE (Figures 7A–C), increased right ventricular wall thickness on HE-stained cardiac sections (Figures 7D,E), higher RVSP (Figure 7F), and an increased Fulton index (Figure 7G). LVEF remained unchanged among groups in this intervention cohort (Supplementary Figure S5). Collectively, these findings support a model in which RvD1 protects against PH at least in part by suppressing pathological GJs-Cx43 formation and GJs-Cx43-mediated intercellular communication.

**FIGURE 7:**
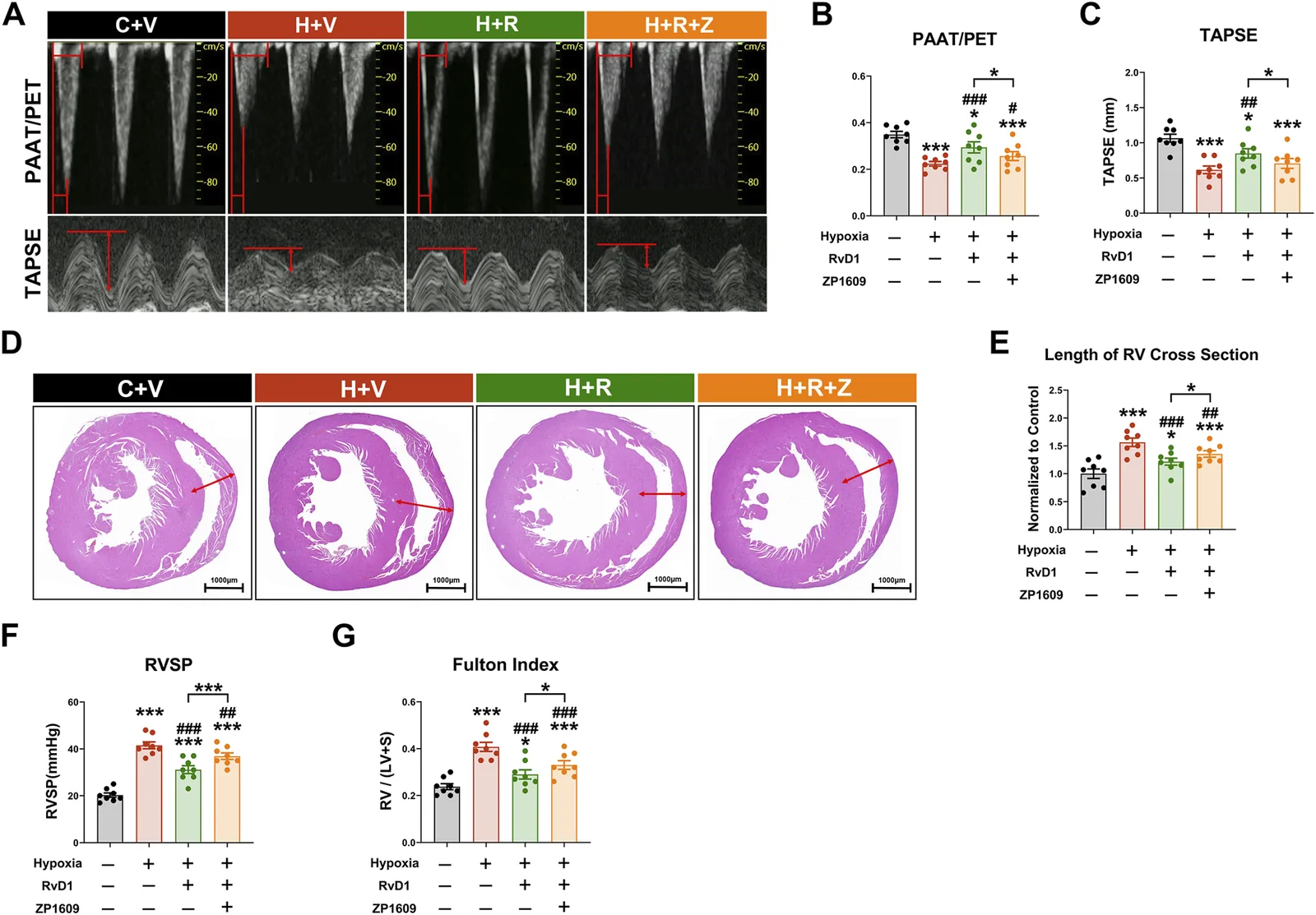
ZP1609 reduces RvD1-mediated improvement in pulmonary hemodynamics and right ventricular remodeling. **(A)** Representative echocardiographic images showing PAAT/PET and TAPSE measurements. **(B)** Quantification of PAAT/PET. **(C)** Quantification of TAPSE. **(D)** Representative HE-stained transverse cardiac sections showing right ventricular wall morphology. **(E)** Quantification of right ventricular cross-sectional wall thickness, normalized to the C + V group. **(F)** Quantification of Right ventricular systolic pressure (RVSP). **(G)** Quantification of Fulton index. Data are presented as mean ± SEM; n = 8 mice per group. C + V: normoxia plus vehicle; H + V: Sugen/hypoxia plus vehicle; H + R: Sugen/hypoxia plus RvD1; H + R + Z: Sugen/hypoxia plus RvD1 and ZP1609. *P < 0.05, **P < 0.01 and ***P < 0.001 *versus* C + V; ^#^ *P* < 0.05, ^##^ *P* < 0.01 and ^###^ *P* < 0.001 *versus* H + V; brackets indicate comparison between H + R and H + R + Z.

## 4 Discussion

This study proposes that excessive GJs-Cx43 formation serves as a pathological communication network in PH. Under physiological conditions, Cx43-mediated gap junctions help maintain coordinated signaling among vascular cells. During Sugen/hypoxia-induced pulmonary hypertension, however, increased GJs-Cx43 enhances intercellular communication, allowing inflammatory and oxidative stress signals to spread over a broader PASMCs network. This amplified signal propagation promotes widespread PASMCs proliferation and contributes to pulmonary vascular remodeling. RvD1 interrupts this process by reducing GJs-Cx43 formation and suppressing gap junctional intercellular communication, thereby limiting PASMCs activation and vascular remodeling. Conversely, ZP1609 restores GJs-Cx43-mediated communication and weakens the protective effects of RvD1. These findings suggest that RvD1 protects against PH, at least in part, by restricting pathological GJs-Cx43-dependent intercellular communication within the PASMCs, as shown in Figure 8.

**FIGURE 8:**
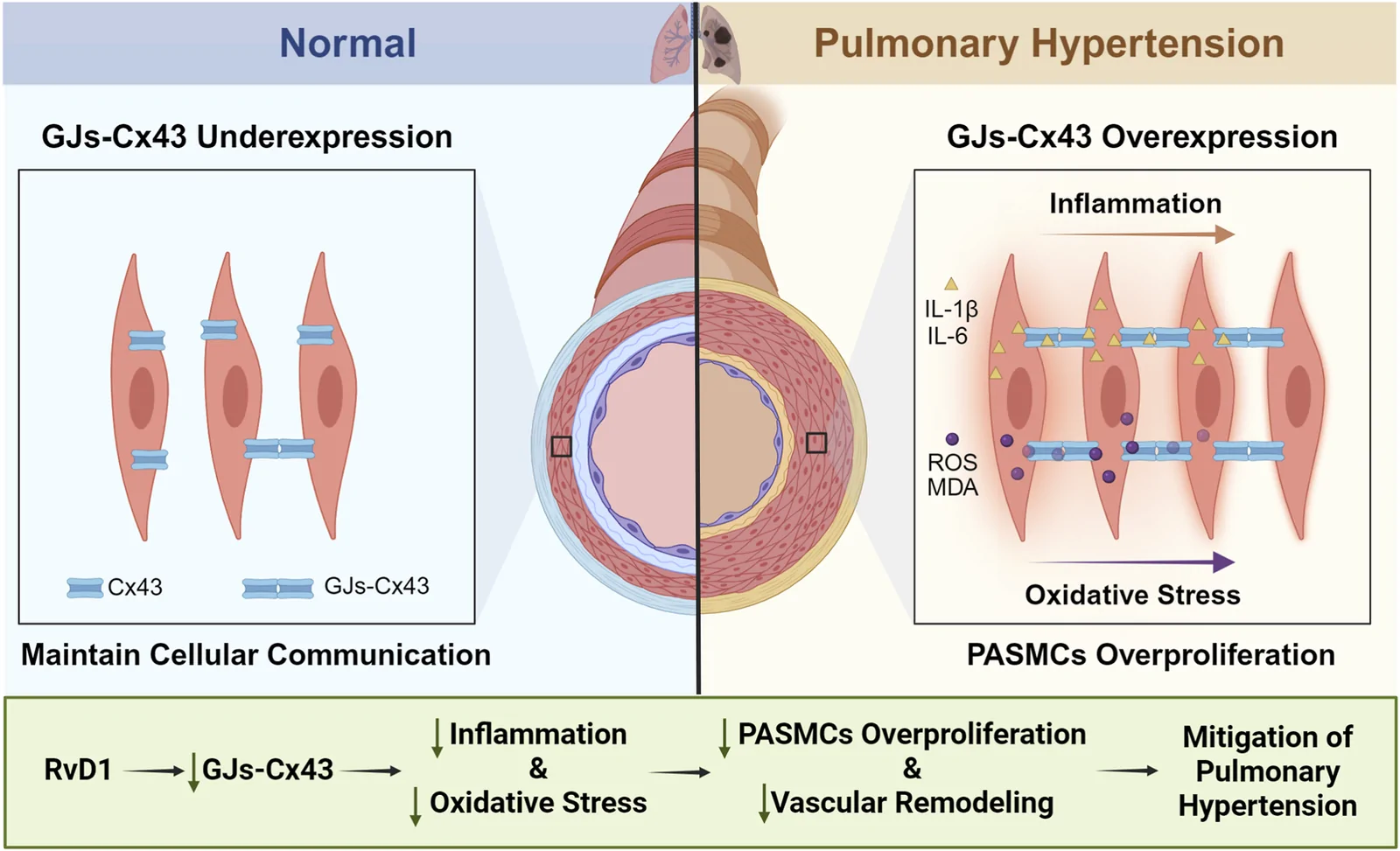
General overview of the main highlights of this study. In PH, excessive GJs-Cx43 formation enhances PASMCs intercellular communication and amplifies inflammation, oxidative stress and PASMCs overproliferation, leading to pulmonary vascular remodeling. RvD1 suppresses GJs-Cx43 formation, reduces inflammatory and oxidative injury, limits PASMC proliferation and mitigates PH.

Our findings refine the role of Cx43 in PH beyond a simple change in total protein abundance. Current evidence supports Cx43 as a PH-relevant molecule across several compartments. Hypoxia-induced Cx43 has been linked to pulmonary arterial fibroblast proliferation and migration (McNair AJ et al., 2020), Cx43 activation contributes to hypoxic PASMCs proliferation and migration (Han X-J, et al., 2021), and Cx43 targeting has shown benefit in hypoxaemic PH models (Bouvard C, et al., 2020). Cx43-related remodeling has also been implicated in right ventricular dysfunction associated with pulmonary arterial hypertension (Alves-Silva JM et al., 2022). These studies establish the importance of Cx43, but most have not resolved which Cx43 configurations is functionally responsible for disease propagation. Our data suggest that the disease-relevant event is not merely more Cx43, but redistribution of Cx43 into a communication-competent GJs-Cx43. This distinction is important because total Cx43 may include non-junctional pools that do not necessarily form intercellular channels, whereas GJs-Cx43 plaques support the direct passage of small signaling molecules between adjacent PASMCs.

One plausible explanation is that excessive GJs-Cx43 acts as a network amplifier in PH. Under physiological conditions, gap junction coupling helps maintain coordinated vascular homeostasis, but pathological hypercoupling may allow stress signals generated in a subset of PASMCs to recruit adjacent cells into the same activated state. Small signaling molecules and second messengers transmitted through gap junctions can synchronize calcium-dependent signaling, redox imbalance and inflammatory activation (Brink PR et al., 2020; Quan Y et al., 2021). Such synchronization would be expected to favor broader PASMCs proliferation and migration than would occur from isolated cell-autonomous injury. In PH, however, excessive GJs-Cx43 may convert focal PASMCs stress into a coordinated remodeling response. Hypoxia can generate local increases in ROS, lipid peroxidation products, inflammatory cytokines in PASMCs (He S-Y et al., 2025; Shi Y et al., 2025). Once PASMCs become strongly coupled through GJs-Cx43, these signals may diffuse or be amplified across neighboring cells, expanding inflammation and oxidative stress beyond the initially injured cell population, thereby amplifying pathological insults and proliferative synchronization across the PASMCs layer. In this context, GJs-Cx43 may act both as a conduit for pathological signal propagation and as a network organizer that enables synchronized PASMCs activation, migration and proliferation within the vascular media. This concept helps reconcile why inflammation, oxidative stress and vascular remodeling often reinforce each other in PH (Jiang G et al., 2024; Poyatos P et al., 2023).

RvD1 is not simply an anti-inflammatory agent, but a pro-resolving mediator that can interrupt self-propagating tissue injury programs (Li et al., 2023). Previous work showed that RvD1 is reduced in PH and that exogenous RvD1 improves pulmonary vascular and right ventricular parameters by inhibiting endothelial-to-mesenchymal transition through an FPR2/Smad-dependent mechanism (Li et al., 2024). Resolvins can also directly modulate vascular tone, including thromboxane mimetic-induced constriction of human pulmonary arteries (Jannaway et al., 2018). The present study extends these observations from endothelial transition and vasoreactivity to PASMCs network behavior, RvD1 reduced GJs-Cx43 formation and suppressed Cx43-mediated intercellular communication, thereby limiting the spread of inflammatory and oxidative stress signals across PASMC networks. Thus, the vascular protective effect of RvD1 in pulmonary hypertension may reflect not only suppression of inflammatory and oxidative responses within individual cells, but also disruption of the intercellular communication pathway that amplifies pulmonary vascular remodeling. If RvD1 were acting only through nonspecific suppression of inflammation, then strengthening GJs-Cx43 coupling would not be expected to meaningfully erode its protective effect. This interpretation does not exclude additional actions of RvD1, including effects on endothelial cells, immune signaling or upstream lipid mediator receptors. Instead, it positions GJs-Cx43 as one convergent node through which multiple injury signals may be organized into a remodeling program. Future work should define how RvD1 regulates GJs-Cx43 assembly, phosphorylation and degradation, processes known to shape gap junction stability and function (Fu et al., 2025; Li et al., 2026; Wang et al., 2025).

An important translational consideration is the selectivity of RvD1-mediated regulation of GJs-Cx43. Because RvD1 was administered intraperitoneally, systemic exposure is expected; however, its tissue distribution, extrapulmonary actions, and systemic safety were not evaluated in the present study. Our findings indicate that RvD1 reduces pathological GJs-Cx43 formation in the SU5416/hypoxia setting, but do not establish that RvD1 directly blocks Cx43 channels or determine its effects on physiological Cx43-mediated communication in healthy tissues. This distinction is relevant because Cx43 is required for normal intercellular coupling in several organs, including the myocardium (Xie et al., 2022; Zhang et al., 2026). In addition, our study specifically targeted Cx43 because it is recognized as a principal and functionally critical connexin isoform mediating pathological remodeling in pulmonary arterial smooth muscle cells (Bouvard C, et al., 2020; Han X-J, et al., 2021; McNair AJ, et al., 2020). Furthermore, GJA1, the gene encoding Cx43, has been reported to be the predominant connexin transcript in cultured PASMCs, although these cells can also express Cx37, Cx40, and Cx45 (Htet M et al., 2021). We did not assess whether RvD1 modulates these connexins or Cx43-independent signaling pathways. Therefore, our data identify pathological GJs-Cx43 as a functionally important component of RvD1-associated protection rather than its exclusive target.

Several limitations should be acknowledged. First, normoxic RvD1-only and ZP1609-only groups were not included. Although a previous rat study using the same daily RvD1 dose reported no apparent effects on basal pulmonary haemodynamic and structural indices (Hiram R et al., 2021), external evidence cannot substitute for contemporaneous controls in the present SU5416/hypoxia mouse model. Accordingly, our conclusions are limited to the effects of RvD1 and ZP1609-sensitive GJs-Cx43 function in the pathological SU5416/hypoxia context. Second, although biochemical fractionation, Cx43 immunofluorescence, dye-transfer assays, and pharmacological enhancement of GJs-Cx43 function consistently support a role for pathological GJs-Cx43 formation, the upstream mechanisms through which RvD1 regulates Cx43 trafficking, plaque assembly, or channel activity remain to be defined. Future studies incorporating normoxic drug-treated controls and targeted mechanistic approaches will further clarify these issues. Third, the inflammatory response was assessed using only two cytokines, IL-1β and IL-6, which limits comprehensive characterization of the broader inflammatory effects of RvD1 in pulmonary hypertension. Future studies incorporating multiplex cytokine profiling will provide a more complete assessment of the inflammatory milieu. Fourth, although our biochemical assays indicated an attenuation of oxidative stress, we did not investigate deeper mechanistic pathways, such as catalase activity or the expression of key oxidative-stress-associated transcription factors like nuclear factor erythroid 2-related factor 2(Nrf2) (Chen S et al., 2022). Exploring the interplay between the Cx43 axis and the Nrf2 antioxidant signaling pathway will be a crucial direction for our future investigations.

## 5 Conclusion

In conclusion, this study identifies pathological GJs-Cx43 formation as a functionally important communication mechanism in PASMCs driven pulmonary vascular remodeling. RvD1 restrains this mechanism and thereby limits the intercellular spread of inflammatory, oxidative and proliferative signaling. The RvD1-GJs-Cx43 axis may therefore represent a pro-resolving strategy for targeting the multicellular communication network that sustains PH vascular remodeling.

## Data Availability

The original contributions presented in the study are included in the article/Supplementary Material, further inquiries can be directed to the corresponding authors.
