# Supplementary material for: Resolvin D1 attenuates pulmonary hypertension by suppressing pathological connexin 43-mediated gap junctional communication in pulmonary arterial smooth muscle cells
Source: Front Pharmacol. 2026 Oct 2;17:1940174. doi: 10.3389/fphar.2026.1940174 (PMC13671970; doi:10.3389/fphar.2026.1940174)
Supplement: Supplementary file 1 [file Supplementaryfile1.docx]

Supplementary Material

## 1.1 Supplementary Figures

**Supplementary Figure S1. LVEF is preserved among experimental groups.**Quantification of left ventricular ejection fraction (LVEF) in C+V, H+V and H+RvD1 mice. Data are presented as mean ± SEM; n=8 mice per group. C+V: normoxia plus vehicle; H+V: Sugen/hypoxia plus vehicle; H+RvD1: Sugen/hypoxia plus RvD1.

**Supplementary Figure S2. Characterization of primary mouse PASMCs.** (A) Representative α-SMA immunofluorescence staining of primary mouse PASMCs. (B) Representative flow cytometry histogram showing α-SMA-positive cells. (C) Quantification of α-SMA-positive cells, indicating high PASMC purity (97.89 ± 1.00 %). Data are presented as mean ± SEM; n=6 per group.

## Supplementary Figure S3. In vitro treatment protocol. Schematic illustration of the in vitro treatment protocol. Primary mouse PASMCs were exposed to hypoxia (2% O2) for 48 h and treated with RvD1 (300 nM) before molecular biology assays.

## Supplementary Figure S4. In vitro treatment protocol for Z1609 intervention. Schematic illustration of the in vitro treatment protocol. Primary mouse PASMCs were exposed to hypoxia (2% O2) for 48 h and treated with RvD1 (300 nM) and/or ZP1609 (1 µg/mL) before molecular biology assays.

**Supplementary Figure S5. LVEF is preserved in the ZP1609 rescue experiment.** Quantification of left ventricular ejection fraction (LVEF) in C+V, H+V, H+R and H+R+Z mice. Data are presented as mean ± SEM; n=8 mice per group. C+V: normoxia plus vehicle; H+V: Sugen/hypoxia plus vehicle; H+R: Sugen/hypoxia plus RvD1; H+R+Z: Sugen/hypoxia plus RvD1 and ZP1609.
